# Enhancing the Recovery of Bioactive Compounds of Soybean Fermented with *Rhizopus oligosporus* Using Supercritical CO_2_: Antioxidant, Anti-Inflammatory, and Oxidative Proprieties of the Resulting Extract

**DOI:** 10.3390/jof8101065

**Published:** 2022-10-11

**Authors:** Fernanda Guilherme do Prado, Maria Giovana Binder Pagnoncelli, Maria Rosa Machado Prado, Marcos Lucio Corazza, Vanete Thomaz Soccol, Gilberto Vinícius de Melo Pereira, Carlos Ricardo Soccol

**Affiliations:** 1 Bioprocess Engineering and Biotechnology Department, Federal University of Paraná, Curitiba 81531-970, Brazil; 2 Department of Chemistry and Biology, Federal University of Technology—Paraná (UTFPR), Curitiba 80230-900, Brazil; 3 Department of Pharmacy, Faculdades Pequeno Principe, Curitiba 80230-020, Brazil; 4 Department of Chemical Engineering, Federal University of Paraná, Curitiba 81531-970, Brazil

**Keywords:** fermented soybean, supercritical fluid extraction, antioxidant activity, phenolic compounds, *Rhizopus oligosporus*

## Abstract

The aim of the present study was to evaluate the use of supercritical CO_2_ combined with cosolvent for the recovery of bioactive compounds of soybean fermented with *Rhizopus oligosporus* NRRL 2710. Soxhlet extractions using seven different organic solvents (*n*-hexane, petroleum ether, ethyl acetate, acetone, ethanol, methanol, and water) were initially performed for comparative purposes. The extracts obtained were characterized by physicochemical, antioxidant, total phenolic, and oxidative proprieties. For the Soxhlet extractions, the highest and lowest yields obtained were 45.24% and 15.56%, using methanol and hexane, respectively. The extraction using supercritical CO_2_ combined with ethanol as a static modifier (scCO_2_ + EtOH) presented, at a high pressure (25 MPa) and temperature (80 °C), a phenolic compound content of 1391.9 μg GAE g^−1^ and scavenging of 0.17 g, reaching a 42.87% yield. The extracts obtained by sCO_2_ + EtOH were characterized by high contents of essential fatty acids (linoleic acid and oleic acid) and bioactive compounds (gallic acid, trans-cinnamic acid, daidzein, and genistein). These extracts also showed a great potential for inhibiting hyaluronidase enzymes (i.e., anti-inflammatory activity). Thermogravimetric analyses of the samples showed similar profiles, with oil degradation values in the range from 145 to 540 °C, indicating progressive oil decomposition with a mass loss ranging from 93 to 98.7%. In summary, this study demonstrated the flexibility of scCO_2_ + EtOH as a green technology that can be used to obtain high-value-added products from fermented soybean.

## 1. Introduction

Soybean (*Glycine max L.)* is one of the main commodities across the world and accounts for 60 percent of the global oilseed production. Its worldwide production in the 2019/2020 harvest was estimated by the United States Department of Agriculture as 335.35 million tons. It is projected that there will be an increase to 362.85 million tons in the current 2020/2021 harvest [1,2]. This trend is strongly correlated with the higher consumption of meat and soy-based foods.

Soybean is an important foodstuff that is used worldwide, popularly known as being one of the most abundant and cheapest sources of high-quality vegetable protein. Some plant sources have bioactive components of interest, such as phenolic compounds, carotenoids, alpha acids, methylxanthines, and vitamins. Phenolics are compounds that consist of flavonoids, phenolic acids, tannins, and other related compounds [3,4]. Soybean is considered the richest source of phenolic compounds in the human diet. They are an important class of phytochemical antioxidants and are used in the food and pharmaceutical industries [5,6].

Fermented soybean products contain phenolic compounds available in the aglycone form and, consequently, they have greater bioactive activities than the “in-nature” soybean. The use of extraction techniques for these high-value-added products increases the concentration of the bioactive compounds [7,8]. Although several studies in the literature have addressed the topic of soy-derived bioactive substances and fermented soybean products [8,9], there is a lack of information on the comparative effects of different extraction methods and operating conditions.

Traditional extraction methods are used to recover many bioactive compounds from plant materials. However, the large number of solvents can result in an expensive and environmentally damaging process due to the use of aggressive and toxic chemicals [10]. Thus, there is an interest in searching for alternative extraction methods which reduce or eliminate the extractive residues [3].

Supercritical carbon dioxide (scCO_2_) appears as a modern method for the extraction of natural compounds with a high added value, as an alternative that encompasses the principles of “green chemistry”, which is inert and non-toxic, cheap, and widely available, with a moderate critical temperature and pressure [11]. Another considerable advantage is gained by the manipulation of the temperature and pressure to adjust the supercritical fluid properties, such as the density, and to increase the solvent selectivity, resulting in different extraction yields and extract compositions [12].

The solubility of long-chain molecules and high-polar compounds in supercritical CO_2_ is limited [13]. This is a disadvantage that can be tackled using a polar co-solvent [14]. The addition of the co-solvent is a useful strategy for enhancing the solvent power, increasing the extraction of polyphenols, and improving the selectivity regarding the extraction yield of compounds from plant matrices (Santos et al., 2017) [15]. Solvents are generally recognized as safe (GRAS) and, according to the American Food and Drug Administration, are preferable for this process. Thus, ethanol has a great advantage and is commonly used, as it is low cost and represents an innocuous solvent in regard to human health and the environment [3].

There are few reports regarding Soxhlet and scCO_2_ + EtOH extractions of soybean fermented with filamentous fungi and the assessment of the biological activities of extracts obtained from the resulting material. Therefore, this study aimed to compare the extracts obtained by Soxhlet extraction with different organic solvents and by extraction with supercritical CO_2_ + ethanol (scCO_2_ + EtOH). The extracts were compared in terms of their extraction yields and general extraction curves (extraction kinetics). The fatty acid (FA) profile, antioxidant activity (AA), total phenolic content (TPC), phenolic compounds (by HPLC), anti-inflammatory activity, and oxidative stability were also evaluated to assess the qualities and the potential commercial applications of the oil.

## 2. Materials and Methods

### 2.1. Microorganism

The fermentation process was performed using a fungus of the *Rhizopus oligosporus* NRRL 2710, belonging to the ARS Culture Collection of the Northern Regional Research Laboratory (NRRL, Peoria, IL, USA). The strains were maintained on potato dextrose agar (PDA) slants at 4 °C.

### 2.2. Substrate Treatment

The substrate used in this study (soybean) was pre-soaked in deionized water containing 5% *v*/*v* acetic acid. After 12 h, the substrate was thermally treated under a flowing steam at 100 °C for 15 min. The excess water was removed, and the substrate cooled to the inoculation temperature at 30 °C.

### 2.3. Fermented Soybean

The substrate, once thermally treated, was inoculated with 10^7^ spores g^−1^ of the dry substrate. The experiments were carried out on perforated trays at 30 °C for 72 h. After fermentation, the samples were crushed in a kitchen blender (Turbo Blender) for 5 min. A fraction was stored in a freezer at −18 °C ± 2 °C for use in the extraction experiments (here named as the wet sample). Another fraction was dried in an oven at 50 °C until a constant weight was obtained (here named as the dry sample). The samples were stored in a freezer at 4 °C for later use. The moisture of the raw material was measured using infrared moisture balance (Bel Engineering, Monza, Italy). All analyses were performed in triplicate.

### 2.4. Soxhlet Extraction

The fermented soybeans were subjected to Soxhlet extraction following the methodology described in the AOCS procedures [16], using a mass of solids to solvent volume ratio of 1:40 (*m*/*v*). Seven different solvents with different polarities were tested to evaluate the extraction yield, as well as the extraction efficiency of the phenolic compounds. Tests were carried out in triplicate for each solvent over 6 h of extraction. All solvents used in this study were of an analytical grade. At the end of extraction, the solvent was removed with a rotary evaporator (RV 10 digital, Ika, Wilmington, USA), and then held at 50 °C in an oven for 24 h to remove any residual solvent. The extract amounts were weighed in an analytical balance to calculate the extraction yield (wt.%) as follows:

(1) $$Extracion\;yield\;\left(\%\right)=\left(\frac{mass\;of\;extrated\;oil\;\left(g\right)}{mass\;of\;sample\;\left(g\right)}\right)\times 100\;$$

where the mass of the sample was expressed on a dry basis, taking into account the moisture content of all the samples used before the extraction.

### 2.5. Supercritical Extraction

The supercritical extraction process was performed using a home-made laboratory scale extraction unit (batch extractor with a 80 cm^3^ internal volume, L = 0.16 m, *ɸ* = 2.52 × 10^−2^ m), as described in [17]. The high-pressure extractor is connected to an ultra-thermostatic bath, and a needle valve is used for the control of the flow of the outlet stream of the extractor. The system also consists of a high-pressure syringe pump (ISCO, model 500D, Lincoln, NE 68504, USA), pressure and temperature sensors, and transducers.

The extractions were performed using a liquid solvent modifier in a semi-batch fixed-bed extraction process, using supercritical CO_2_ as the solvent and ethanol as the liquid solvent, named as scCO_2_ + EtOH. The experimental procedure consisted of soaking the raw material in the ethanol solvent using a 1:1 (*w*/*w*) ratio, keeping the mixture static for 60 min. Previously, different concentrations of ethanol were tested (data not shown). The chosen ratio was 1:1 (*w*/*w*), again following previous literature results [1,2,3]. The mixture was loaded into the extraction vessel, and CO_2_ was applied until the pressure reached the extraction condition. Then, the mixture containing the raw material (fermented solids) with ethanol and CO_2_ was maintained for a confinement period (static extraction step) of 60 min to ensure the thermal and mechanical equilibration before the start of the dynamic extraction stage.

The scCO_2_ + EtOH extractions were performed based on a 2^2^ factorial experimental design with a center point, aiming to evaluate the effects of the temperature, ranging from 40 to 80 °C, and pressure, ranging from to 15 a 25 MPa, on the extraction yield and the quality of the extracts. The compressed solvent (supercritical CO_2_) was pumped at a constant flow rate of 2.0 ± 0.3 cm^3^ min^−1^.

The extracts were collected in amber glass vessels and their weight was determined at each extraction time interval of 5 min.

### 2.6. Determination of the Fatty Acid Profile

The fatty acid profile of the fermented soybean oil was analyzed using a Shimadzu chromatograph (GC 2010 *Plus*), a capillary column (SH-Rtx-Wax (Shimadzu, Kyoto, Japan): 30 m × 0.32 mm × 0.25 μm), a flame ionization detector (FID), and the split injection mode (1:10). The injector and detector temperatures were 240 °C and 250 °C, respectively. The oven temperature was programmed to start at 100 °C for 5 min, followed by an increase up to 240 °C at a rate of 4 °C min^−1^, and we maintained this temperature for 5 min. The carrier gas was helium at 32.5 cm^3^ min^−1^. The samples were prepared according to the official method [16] used to convert triacylglycerol and free fatty acids of samples into fatty acid methyl esters (FAMEs). FAMEs were identified by comparison with the retention times of the standard mixture FAMEs (Supelco, MIX FAME 37, St. Louis, MO 63103, USA). The quantification of the fatty acids was conducted using the normalization area procedure. Results were expressed as the percentage of each fatty acid present in the sample.

### 2.7. Determination of the Total Phenolic Content

The total phenolic content of the extracts was determined by the Folin–Ciocalteu method [18] with modifications. This test is based on the oxidation of phenolic groups with phosphomolybdic and phosphotungstic acids. After oxidation, the absorbance of the green-blue complex formed was measured at 760 nm. In a test tube, 0.1 mL of the sample was mixed with 0.5 mL of Folin–Ciocalteau reagent (10% *v*/*v*) and 0.4 mL of sodium carbonate (7.5% *w*/*v*). The samples were shaken for 1 h and protected from light (reaction time). Ethanol was used as a blank. The samples were analyzed in triplicate. A standard curve prepared using gallic acid monohydrate was used to calculate the total phenol content, which was expressed as the gallic acid equivalent of the extract in µg g^−1^ (Singleton, 1965).

### 2.8. Measurement of the DPPH Radical Scavenging Activity

The antioxidant activity of the extracts was assessed based on the radical scavenging effect of the stable DPPH free radical activity (1,1-diphenyl-2-picryhydarzyl) (St. Louis, MA, USA), as described by [19]. A methanol solution of DPPH (0.004% *w*/*v*) was prepared. One milliliter of methanolic DPPH solution was added to 250 µL of the sample extracts at different concentrations (50; 100; 150; 200; 250 V/V). After incubation in the dark at an ambient temperature for 30 min, the absorbance was measured at 517 nm (spectrum band, UV-VIS). Ethanol solution without the sample was used as a control. The IC_50_ value (index representing the concentration of the antioxidant, which can reduce the free radicals by 50%) was obtained using a standard curve. The inhibition rate (%) was calculated using the following equation:

(2) $$\mathrm{Inibition}\;\left(\%\right)=\left(\frac{A_{\mathrm{control}\;}-{\;A}_{\mathrm{extract}}}{A_{\mathrm{control}}}\right)\times 100$$

where:

A_control_ = DPPH absorbance (diluted with ethanol solution);

A_extract_ =DPPH + sample extract absorbance (diluted with ethanol solution).

The antioxidant activity was plotted (percent) against the log absorbance values, generating a straight line that we used to calculate the half-inhibitory concentration (IC_50_) in g g^−1^. The anti-free radical activity (expressed as IC_50_) is defined as the number of antioxidant substances needed to reduce the initial concentration of DPPH by 50%. The higher the power of the anti-free radical is, the more effective the antioxidant and, consequently, the lower the IC_50_ value will be [19]. Trolox (6-hydroxy-2,5,7,8-tetramethylchroman-2-carboxylic acid) (Sigma-Aldrich, USA) was used as a standard, prepared in concentrations ranging from 1 to 100 μm of trolox mL^−1^. The results were expressed by comparing the equivalence of the antioxidant potential of the extracts to the standard potential.

### 2.9. High-Performance Liquid Cromatography of the Phenol Contents (HPLC)

The phenolic content of the extracts was analyzed by high-performance liquid chromatography using an Agilent Technology 1200 Series system coupled with a diode array detector (DAD) at wavelengths of 235, 260, 275, 280, 290, 311, 357, and 370 nm and a scanning range from 190 nm to 600 nm. A Zorbax Elipse XDB—C 18 (4.6 × 150 mm, 5—micron) column was used at a 0.7 mL min−1 flow. The mobile phase utilized was methanol and 2.5% acetic acid (50:50 *v*/*v*). Chlorogenic acid, caffeic acid, ferulic acid, tocopherol, genistein, daidzein, trans-cinnamic acid, catechin, rutin, p-coumaric acid, gallic acid, resveratrol, and epicatechin (SIGMA Aldrich, EUA, St. Louis, MO, USA) were used as standards. To obtain the calibration curves, all standard reagents (SIGMA Aldrich, EUA) were solved in mobile phase and used at 1, 2, 5, 8, and 10 ppm. The injection volume was 10 µL and the run time was 36 min. The resulting chromatograms values were mapped on graphs, and a linear equation was used to calculate the phenolic compound content of the samples. The samples were microfiltered trough a hydrophilic membrane GV (Durapore) formed of polyvinylidene difluoride (PVDF), with a pore size of 0.22 μm. The results were shown in μg g^−1^ values of the phenolic compounds identified.

### 2.10. Evaluation of the Anti-Inflammatory Activity

To evaluate the anti-inflammatory activity, with the inhibition of the hyaluronidase enzyme, an in vitro method was used, according to [20]. The anti-inflammatory potential was measured at concentrations ranging from 0.5 to 2.5 μg mL^−1^. For the sample analysis, 50 μL of extract was placed in different concentrations with 0.5 mL of potassium salt of hyaluronic acid (Sigma-Aldrich, St. Louis, MO, USA) (1.2 mg hyaluronic acid per mL of 0.1 M acetate buffer, pH 3.6, containing 0.15 M NaCl) in a reaction tube. The control tube consisted of the same reagent used for the test tubes without ExPP. All the tubes were incubated for 5 min at 37 °C and, after that, 50 μL of the enzyme hyaluronidase (350 units of the enzyme hyaluronidase type IV-S from bovine testes, Sigma-Aldrich, St. Louis, MO, USA, dissolved in the same buffer substrate at concentration 6.5 mg/mL) was added, and the samples were incubated at 37 °C for 40 min. The reaction was ended by adding 10 L of 4 N sodium hydroxide solution and immediately adding 0.1 mL of 0.8 M potassium tetraborate into the reaction mixture and incubating it in a boiling bath for 3 min. After the incubation time, we added 3 mL of 4-dimethylaminobenzaldehyde (DMAB) (10% solution in glacial acetic acid containing 12.5% 10 N hydrochloric acid) to the reacted mixture and incubated the solution at 37 °C for 20 min. Next, we measured the samples using a spectrophotometer (SP 2000 UV Spectrum) at 585 nm. DMSO was used as positive control due to its ability to completely inhibit the hyaluronidase enzyme. Propolis, a natural anti-inflammatory agent, was also included as a positive control. The results were expressed as the ability to inhibit the hyaluronidase enzyme in percentages.

### 2.11. Determination of the Oxidative Stability

The thermogravimetric curves were obtained using the Perkin–Elmer thermogravimetric analyzer TGA 4000 model. The temperature ranged from 30 °C to 800 °C and the heating rate was 10 °C min^−1^. A dynamic atmosphere of synthetic air was used at a flow rate of 50 mL min^−1^. Ceramic pots and initial sample masses ranging from about 8 to 10 mg were used in the apparatus.

### 2.12. Statistical Analysis

The results of the analysis were subjected to statistical variance analysis and the post test was chosen according to the normality of the data obtained, e.g., we used the Tukey post test for data which exhibited normality. The program PRISMA^®^ (GraphPad Prism 5 for Windows, version 5.4) was used. The averages of the data are presented in graphs and the standard deviation is shown by the error bars.

## 3. Results and Discussion

### 3.1. Soxhlet Extraction

Table 1 shows the Soxhlet extraction values for different organic solvents. The highest extraction yields were 44.10% and 45.24% for ethanol and methanol, respectively, suggesting that the target compounds of the studied matrix have intermediate to high polarity. Thus, the higher the solvent polarity was, the higher the extract yield was found to be.

The extractions were performed with dried and non-dried raw materials, in which the residual moisture content was 23% and 73.5%, respectively. Extractions using solvents of medium to high polarity were performed using a dry sample, The results in Table 1 showed yields of 25.48% and 32.84% for ethanol and methanol, respectively. It was observed that the drying process enabled a significant increase in the extraction yield of up to 18.62% using ethanol. Thus, the moisture levels in the raw material influence the extraction kinetics and yield, since water is an interfering factor in the solvent’s penetration of the solid matrix and in the diffusion of the oil [21]. Previous studies also reported the optimization of the extraction using a lower moisture content, stating that the drying process facilitates the contact between the solvent and the solute to be extracted [21,22,23].

Although the use of water as a solvent in the Soxhlet extraction results in a higher yield, the high boiling temperature can lead to the degradation of bioactive compounds, such as polyphenols and flavonoids [24]. Among the organic solvents, ethanol, in addition to providing a better extraction due to its high polarity, also has lower toxicity and poses fewer risks to human health. Thus, ethanol is more desirable than methanol, and it was, therefore, selected as the liquid solvent for the further extraction procedures.

### 3.2. Extraction by Compressed Solvents

Initially, an experiment was performed using supercritical CO_2_ at 80 °C and 25 MPa, whereby the extraction yield obtained was extremely low (<1%). Therefore, we chose to perform all the additional extractions using the combined solvents scCO_2_ + ethanol. In addition, a preliminary pretreatment of the feedstock with ethanol was performed to promote and improve the contact between the solvent and the matrix before the compressed solvent extractions. After some preliminary testing, it was observed that 60 min of contact was a suitable condition for this pretreatment. The raw material–ethanol contact can alter the matrix by swelling, thereby prompting the transport of the analyte from the interstitial pores to the surface and, then, to the bulk phase [25].

A 2^2^ factorial design with a central point was performed to verify the influences of the process parameters, such as the pressure and temperature. The results obtained are shown in Table 2.

All scCO_2_ + ethanol experiments were performed using a 60 min static extraction time (confinement period). It was observed that the highest extraction yield (42.87 wt.%) was obtained under the highest conditions of pressure and temperature, i.e., 25 MPa and 80 °C. The comparison of runs 2 and 4 showed that changing the pressure, at 80 °C, increased the yield to 15 percentual points (p.p.). In addition, after varying the temperature at a fixed pressure of 25 MPa, an increase in yield by 12 p.p. was observed. In general, the addition of ethanol as a polarity modifier to scCO_2_ proved to be an efficient approach to raising the extraction yield levels, compared to Soxhlet extraction (Table 1), but using a lower amount of ethanol.

**Figure 1:**
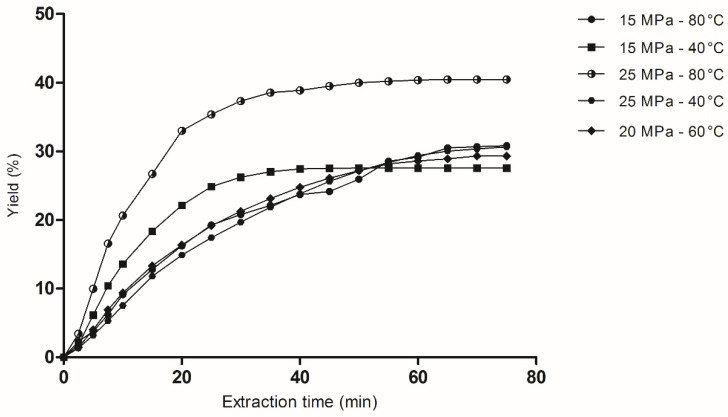
Overall extraction curves for dried fermented soybean using scCO_2_ + EtOH as a solvent.

To better understand the extraction using scCO_2_ + EtOH, the general extraction curves are provided in Figure 1. The results showed that the yields obtained were significantly affected by the operational parameters studied. The pressure and temperature parameters had positive effects on the yield because, and at higher levels (80 °C and 25 MPa), they fostered higher yield values. The values obtained under different conditions presented a significant difference (*p* > 0.05).

The lowest yield (27.56%) can be observed when using the lowest pressure (15 MPa) and highest temperature (80 °C). Pressure is the parameter that positively influences the yield of scCO_2_, so that the increase reflects the increases in density, viscosity, and the solvation power of CO_2_ [26,27]_._ Therefore, the lower yield is probably a reflection of the low solubility of the extract, since the lower CO_2_ density results in low mass transfer rates during extraction [28,29].

Analyzing all the extraction curves in Figure 1, it was observed that there were high initial extraction rates for up to 40 min and a slope of the curve in the subsequent minutes. This indicates that there was first a period of a dynamic extraction rate, in which the outer surface of the particles is covered with the easily accessible and available solute. Then, the scCO_2_ ceases from operating as a carrier and starts operating as an extraction agent, and the mass transfer occurs by diffusion within the solute particles, resulting in a drop in the extraction rate [30].

Comparing both abovementioned techniques, Soxhlet extraction using ethanol and methanol as solvents showed a higher yield (44.10 and 45.24%, respectively) compared to scCO_2_, where the maximum yield obtained under different extraction conditions was 42.87%. However, in terms of time, scCO_2_ extraction is less time consuming than Soxhlet extraction.

### 3.3. Fatty Acid Profile

Fatty acids are key components of the phospholipids in cell membranes, playing important roles in various cellular functions, including metabolism and immune responses [31]. The inclusion of oleic acid in the diet is recommended because of its protective effects against cardiovascular and neurodegenerative diseases. Linoleic acid, characteristic of soybeans and present in most of the oils obtained, has gained prominence in recent decades due to its biological and physiological benefits, including anti-carcinogenic [32], anti-obesity [33], and antidiabetic [34] effects, as well as bone-formation-promoting properties [35].

Table 3 shows the fatty acid (FA) profiles of the fermented soybean oil samples obtained using the different methods studied. The FA contents were similar, despite the different extraction conditions and solvents used.

According to the results, the main fatty acids present in all the oil samples were linoleic acid and oleic acid. In the extracts obtained by Soxhlet extraction, these values ranged from 45.22 to 51.92% and from 29.96 to 33.41%, respectively. The content of unsaturated fatty acids (C18:3, C18:1, and C18:2) accounts for about 82.70% of the total fatty acids present in the fermented soybean oil. These values suggest that the extract obtained can be used as an attractive option in the functional food market.

In a study by Man et al., [36] soybean oil, extracted with *n*-hexane by the Soxhlet method, had a predominant content of linoleic acid (range 49.56–55.83%), followed by oleic acid (21.77–26.55%) and palmitic acid (10.50–11.82%). In addition, the content of unsaturated fatty acids was approximately 75%. A comparison between the soybeans of different cultivars, tegument colors, and years of harvest showed that the results, in regard to the fatty acid content, did not present significant differences.

A similar result was observed in a study [37] that aimed to analyze fluctuations in the composition of soybean fatty acids over two years of storage at room temperature. The obtained fatty acid profile ranged from 49.2–56.8% of linoleic acid, 19.7–23.1% of oleic acid, and 9.4–12.4% of palmitic acid, still presenting a total average content of unsaturated fatty acids of >75%. Small changes in the composition of the fatty acids did not show significant variation [37].

Comparing the fatty acid profiles of the oils obtained in this study with the fatty acid profile of the soybean oil reported in *Nature* [36], increases of 2.31% in the oleic acid content and 7.7% in unsaturated fatty acids were observed. Therefore, it is not possible to state that the fermentative process had a positive influence on the composition of FA.

Tian et al. [38] performed a comparative analysis of the fatty acid compositions of unfermented and fermented soybean extracts extracted by SC-CO_2_. While the oleic acid content decreased from 22.35% to 18.59% after the fermentation process, the levels of palmitic acid and α-linolenic acid showed increases ranging from 12.13% to 15.41% and 7.30% to 8.43%, respectively. Comparing this aforementioned study with the present work, it is not possible to state that the fermentation process and the addition of ethanol as a co-solvent are factors that affect the fatty acid profiles of the extracts.

### 3.4. Total Phenolic Content (TPC)

Table 4 presents the total phenolic contents (TPC) for both extraction techniques. In Soxhlet extraction, the values ranged from 132.10 ± 0.01 µg GAE g^−1^ to 1305.6 ± 0.03 µg GAE g^−1^. The best results were obtained from fermented soybeans when using solvents with higher polarity, such as ethanol and water, yielding values of 962.9 µg GAE g^−1^ ± 0.02 and 1305.6 µg GAE g^−1^ ± 0.03, respectively. The results highlight a significant difference (*p* < 0.05). This shows that the polarity of the solvent is a determining factor for the obtainment of phenolic compounds in the extract, indicating that a higher polarity enables one to extract a greater number of polar compounds.

The quantification of the total phenolic compounds contained in the extracts obtained by scCO_2_ + EtOH was performed, and the values obtained ranged from 1058.2 ± 0.01 μg GAE g^−1^ to 1391.9 ± 0.09 μg GAE g^−1^ (Table 4), with a statistical difference (*p* > 0.05). The most efficient conditions for the extraction of such compounds were a high pressure and temperature (1391.9 μg GAE g^−1^). Thus, the pressure had a positive influence on the extraction, since a higher content of total phenolics was observed in the extracts from runs 3 and 4 (25 MPa).

The obtained TPC values reveal that the extracts are beneficial for human health, since according to the regulation established by the EU, no. 432/2012, the limit of 0.25 mg phenolics per g of oil is established as yielding benefits [39].

### 3.5. Antioxidant Potential

Considering that compounds with antioxidant potential are linked to human health benefits and their correlation with the total phenolic compounds, the antioxidant properties of the extracts were quantified. IC_50_ values represent the concentration of a sample that is minimally effective as an inhibitor. According to the values reported in Table 4, in Soxhlet extraction, values vary from 2.93 ± 0.47 g g^−1^ to 0.55 ± 0.84 g g^−1^. The extract with the highest antioxidant potential (water) showed a DPPH radical inhibition potential of 81.27% and 962.66 µm of Trolox g^−1^. This behavior showed that the solvents of intermediate and high polarity used in the extractions favored the solubilization of compounds with antioxidant activities that are detectable using the DPPH method.

For the scCO_2_ + EtOH extracts, the highest antioxidant activity, corresponding to an IC_50_ of 0.17 g g^−1^ and 984 μm of Trolox g^−1^, was observed in extracts obtained under high-temperature conditions (80 °C) and pressure (25 MPa). These extracts were able to inhibit 94.09% of the DPPH radical, which is significantly higher than the other treatments (*p* < 0.05). These results confirm the correlation between the phenolic compounds and the antioxidant activity, as previously reported by other authors [40]. Analyzing these results, it can be stated that no tendency of the antioxidant activity concerning the pressure or temperature was observed for any of the extracts obtained.

When comparing the samples subjected to different extraction techniques, scCO_2_ + EtOH proved to be a viable approach, since it enabled the extraction of higher-quality extracts compared to those obtained by Soxhlet extraction with an organic solvent. Comparing the extracts obtained from the fermented soybean using scCO_2_ + EtOH, the antioxidant potential was 3.23 times higher than that of the extracts obtained by Soxhlet.

Given these results, the further analysis conducted in this study focused on the extracts obtained by sCO_2_ + EtOH.

### 3.6. High-Performance Liquid Cromatography of the Phenol Contents (HPLC)

The chromatographic analysis resulted in the identification of four phenolic compounds (Table 5). This analysis revealed the presence of the same phenolic compounds in all the extracts tested, including gallic acid (benzyl 7-hydroxy-2,2-diphenyl-1,3-benzodioxole-5-carboxylate), trans-cinnamic acid ((E)-3-phenylprop-2-enoic acid), genistein (5,7-dihydroxy-3-(4-hydroxyphenyl)chromen-4-one), and daidzein (7-hydroxy-3-(4-hydroxyphenyl)chromen-4-one), with variations in the retention time (Rt).

Phenolic acids are non-flavonoid compounds that can be distinguished by their chemical structure [41]. The chromatographic analysis revealed the presence of benzoic acid derivatives, such as gallic acid, and derivatives of cinnamic acid, such as trans-cinnamic acid. The values of gallic acid ranged from 414.5 to 457.2 μg g^−1^ among the extracts obtained under different conditions, and trans-cinnamic acid ranged from 518.1 to 521.4 μg g−^1^.

The presence of aglycone isoflavones, such as daidzein and genistein, was also observed. The values obtained varied from 565.9 to 606.1 μg g^−1^ of daidzein and 593.1 to 625.3 μg g^−1^ of genistein. The authors of [42], in a study of the presence of bioactive compounds in soybean, reported daidzein values of 77.18 μg g^−1^ and genistein 332.02 μg g^−1^. The authors of [43], in studies using fermented soybean by-product (okara) fermented with *Saccharomyces cerevisiae*, also reported the presence of aglycone isoflavones. The presence of these compounds is expected, since the fermentation of grains by microorganisms that produce β-glucosidase, such as *Rhizopus* spp., promotes the biotransformation of glycosidic isoflavones.

### 3.7. Evaluation of the Anti-Inflammatory Activity In Vitro

The anti-inflammatory potential of the extracts obtained by the scCO_2_ + EtOH method under different conditions was evaluated by assessing the inhibitory activity of the hyaluronidase enzyme (HIA). This enzyme is directly involved in inflammatory processes, since it acts on the extracellular matrix of the tissues, increasing the permeability and facilitating its access by pathogens [44].

**Figure 2:**
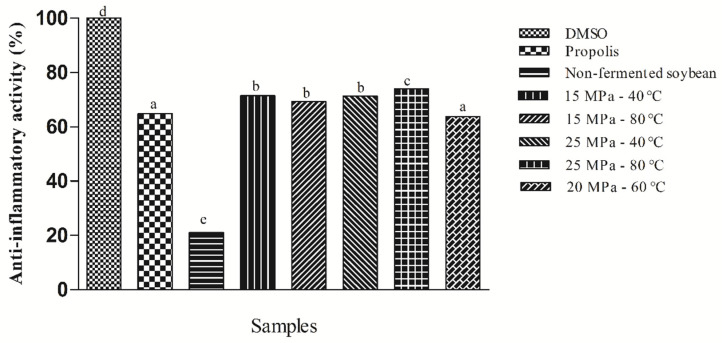
Determination of the anti-inflammatory activity of the samples extracted by scCO_2_ + EtOH under different conditions of temperature and pressure in comparison with different positive controls. DMSO: positive control capable of completely inhibiting the enzyme hyaluronidase (HIA). Propolis: commercial anti-inflammatory. Different letters indicate a significant difference (*p* < 0.05).

The results are shown in Figure 2. The samples showed a significant difference (*p* < 0.05). The extracts obtained by scCO_2_ + EtOH showed potential for the inhibition of the hyaluronidase enzyme in the range of 63.83% to 74.0%. These results were compared with the dimethyl sulfoxide control (DMSO), since it has a complete HIA inhibition capacity, and with the propolis control, a commercial anti-inflammatory. The extracts obtained by scCO_2_ + EtOH showed 9.15% greater anti-inflammatory potential than the tested commercial control, which was able to inhibit 64.85% of the enzymes. It is possible to observe that, as with the previous results, the extract with the highest inhibitory capacity for inhibiting the hyaluronidase enzyme was obtained under the conditions of high pressure and temperature (25 MPa and 80 °C).

A study reported in [8] investigated the anti-inflammatory activity of fermented and unfermented soybean extracts. The results showed that the alcoholic extract obtained in the 72 h fermentation process showed a maximum inhibition capacity of 70.75%, corresponding to an increase in the activity by 3.36 times compared to the “in-nature” extract (21.03%).

The anti-inflammatory potential of the tested extracts may be associated with the presence of total phenolic compounds within them. Several studies have demonstrated that plant-derived polyphenols, especially flavonoids, have potential anti-inflammatory activities in vitro and in vivo [45]. Isoflavones, found mainly in soybean, are especially widely studied because they act as an anti-inflammatory agent, since genistein negatively regulates the signal transduction events induced by cytokines in the cells of the immune system [46].

### 3.8. Oxidative Stability

**Figure 3:**
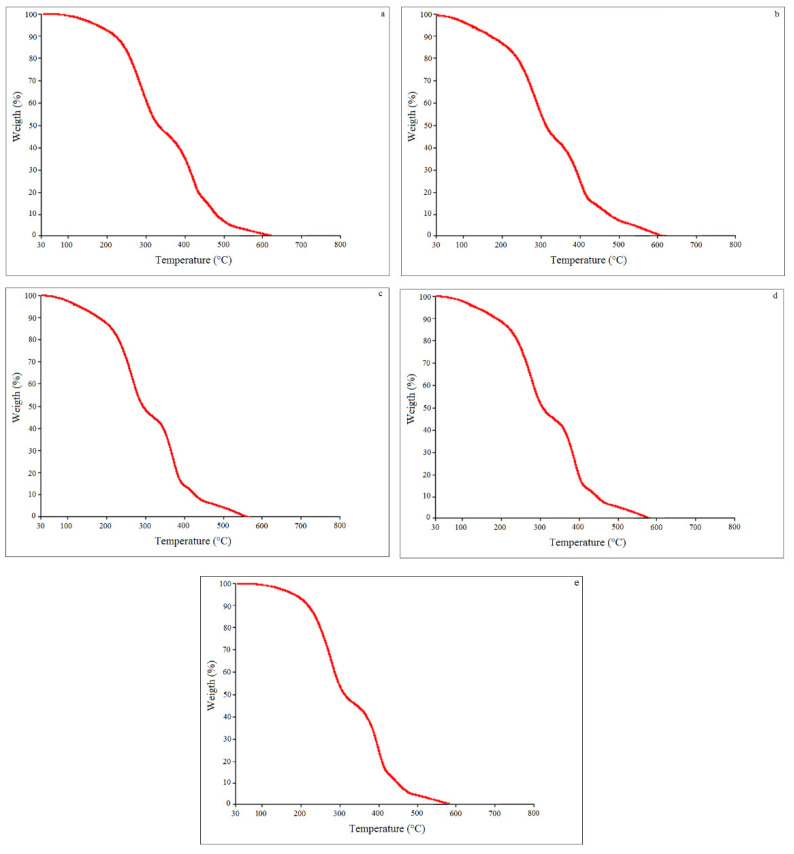
TGA curves of the samples extracted by scCO_2_ + EtOH in an atmosphere of synthetic air under different conditions of temperature and pressure. (**a**) 15 Mpa—40 °C; (**b**) 15 MPa—80 °C; (**c**) 25 MPa—40 °C; (**d**) 25 MPa—80 °C; (**e**) 20 MPa—60 °C.

Thermogravimetric analyses were performed and allowed us to investigate the thermal degradation of the samples in a synthetic air atmosphere and under a controlled temperature. Figure 3 shows the TG curves of the five samples analyzed.

The thermogravimetric curves of the oils showed similar profiles in terms of the mass loss (range from 93.0 to 98.7%) between 145 and 540 °C, relative to the volatilization and/or decomposition of the oil.

The curves indicate the progressive decomposition of the oil. It was verified that both the samples obtained under high-temperature conditions presented the first mass loss peak in the range of 145 °C (7.66 and 4.83%), presenting progressive losses up to 540 °C. These were slightly more stable compared to the oils obtained under low-temperature conditions.

The authors of [47], in studies on different vegetable oils, verified the loss of the soybean oil mass during three thermal events, with the first around the temperature of 379 °C, with a loss of mass of 86.66%. The authors of [48], in studies of soybean biodiesel, observed a mass loss of 99% during only one thermal event in the temperature range of 110 to 250 °C. In this study, all the oils presented up to nine thermal events up to the time of total degradation, reaching mass loss values of 89% at intervals ranging from 412.5 to 430.5 °C.

## 4. Conclusions

In this work, different extraction techniques were used to obtain soybean extracts fermented with *Rhizopus oligosporus* NRRL 2710. The use of solvents with greater polarity resulted in higher extraction yields of bioactive compounds with an antioxidant power, which were rich in phenolic compounds. The extraction efficiency using scCO_2_ + EtOH was increased under a higher pressure and fixed temperature. The most efficient extraction conditions were observed at 25 MPa and 80 °C. An important correlation was observed between the total phenolic content and the antioxidant capacity of the extracts. The fatty acid contents were not significantly affected by the extraction technique used. The main fatty acids reported included linoleic (51.92% and 41.93%) and oleic (33.41% and 37.25%) acid for the Soxhlet and scCO_2_ + EtOH extracts, respectively. The extracts obtained using the supercritical fluid showed anti-inflammatory activity, with great potential for the inhibition of the hyaluronidase enzyme (74%). In addition, important bioactive compounds were detected, including gallic acid and trans-cinnamic acid. Extraction using scCO_2_ + EtOH allowed for a significant improvement in the soybean extracts fermented with *Rhizopus* spp., demonstrating the technical feasibility of the process and enabling the recovery of molecules with great bioactive potential for application in the pharmaceutical and food industries.

## Figures and Tables

**Table 1:** Results of the extraction yield (%) using the Soxhlet extraction process for dried and whole (without drying) fermented soybean samples using different solvents.

				Extraction Yield (wt%)	
Run	Solvents	T (°C) *	Polarity	Dry Sample	Wet Sample
S1	Hexane	68.0	0.0	15.56 ± 4.02 ^c^	-
S2	Petroleum ether	34.61	0.1	29.05 ± 0.51 ^a^	-
S3	Ethyl acetate	77.5	4.3	32.04 ± 2.14 ^ab^	-
S4	Acetone	56.0	5.4	34.07 ± 0.68 ^ab^	19.76 ± 1.05 ^ab^
S5	Ethanol	78.5	5.2	44.10 ± 1.99 ^b^	25.48 ± 4.25 ^bc^
S6	Methanol	64.7	6.6	45.24 ± 3.70 ^b^	32.84 ± 3.70 ^c^
S7	Water	100.0	10.2	23.66 ± 4.32 ^a^	14.20 ± 2.82 ^a^

* Boiling point of the solvents. Means followed by the same letter do not differ statistically (*p* > 0.05).

**Table 2:** Experimental conditions and extraction yield using scCO_2_ + EtOH as a solvent considering dried fermented soybean raw material.

Run (M_RM_)	Solvent	P (MPa)	T (°C)	Time (min)	Extraction Yield (wt%)
1	CO_2_ + EtOH	15	40	75	30.81
2	CO_2_ + EtOH	15	80	75	27.56
3	CO_2_ + EtOH	25	40	75	30.65
4	CO_2_ + EtOH	25	80	75	42.87
5	CO_2_ + EtOH	20	60	75	29.32

**Table 3:** Fatty acid composition of fermented soybean extracts obtained by Soxhlet extraction using different solvents and scCO_2_ + EtOH under different conditions.

Run	Extraction Condition	Composition (%)						
	Soxhlet	Palmitic (C16:0)	Stearic (C18:0)	Oleic (C18:1)	α-Linolenic (C18:3)	Linoleic (C18:2)	Gondoic (C20:1)	Tricosanoic (C23:0)
S1	Hexane	11.35 ^d^	4.33 ^b^	31.86 ^e^	5.47 ^c^	46.32 ^f^	-	0.67 ^a^
S2	Petroleum ehter	12.11 ^d^	4.49 ^b^	30.75 ^e^	5.49 ^c^	46.04 ^f^	0.51 ^a^	0.61 ^a^
S3	Ethyl acetate	11.53 ^d^	4.17 ^b^	30.94 ^e^	5.62 ^c^	46.52 ^f^	0.59 ^a^	0.62 ^a^
S4	Acetone	11.58 ^d^	4.24 ^b^	30.83 ^e^	5.59 ^c^	46.66 ^f^	0.45 ^a^	0.64 ^a^
S5	Ethanol	13.27 ^d^	4.88 ^b^	29.96 ^e^	5.23 ^c^	45.22 ^f^	0.62 ^a^	0.81 ^a^
S6	Methanol	12.37 ^d^	4.50 ^b^	30.21 ^e^	5.64 ^c^	46.14 ^f^	0.45 ^a^	0.69 ^a^
S7	Water	14.68 ^a^	-	33.41 ^b^	-	51.92 ^c^	-	-
	scCO_2_ + EtOH							
1	15 MPa:40 °C	11.27 ^d^	4.57 ^c^	37.34 ^e^	4.27 ^b^	41.45 ^f^	0.51 ^a^	0.59 ^a^
2	15 MPa:80 °C	11.28 ^d^	4.69 ^c^	37.24 ^e^	4.17 ^b^	41.38 ^f^	0.59 ^a^	0.64 ^a^
3	25 MPa:40 °C	10.98 ^d^	4.55 ^c^	37.25 ^e^	4.26 ^b^	41.93 ^f^	0.50 ^a^	0.54 ^a^
4	25 MPa:80 °C	11.31 ^d^	4.63 ^c^	37.17 ^e^	4.23 ^b^	41.42 ^f^	0.55 ^a^	0.68 ^a^
5	20 MPa:60 °C	11.36 ^d^	4.71 ^c^	37.13 ^e^	4.27 ^b^	41.37 ^f^	0.56 ^a^	0.61 ^a^

Different superscripts indicate a significant difference (*p* < 0.05).

**Table 4:** Results of the phenolic compounds (µg GAE g^−1^), with antioxidant activity expressed by the inhibitory concentration (IC_50_), radical scavenging activity expressed by the DPPH (%), and the capacity equivalent to the radical Trolox (µm Trolox/g) of the Soxhlet and scCO_2_ + EtOH extraction.

Run	Extraction Condition	Total Phenolic Compounds (µg GAE g^−1^)	Antioxidant Activity		
	Soxhlet		IC_50_ (g g^−1^)	Inibition DPPH (%)	µm Trolox/g
S1	Hexane	132.1 ± 0.02 ^a^	2.91 ± 0.47 ^a^	18.25 ± 0.99 ^a^	*
S2	Petroleum ehter	423.8 ± 0.02 ^b^	1.75 ± 1.65 ^e^	29.68 ± 0.74 ^b^	*
S3	Ethyl acetate	581.7 ± 0.11 ^d^	2.93 ± 0.84 ^a^	17.53 ± 0.20 ^a^	37.48 ± 0.01 ^a^
S4	Acetone	904.7 ± 0.03 ^e^	0.79 ± 0.27 ^c^	61.28 ± 0.51 ^f^	616.0 ± 0.01 ^d^
S5	Ethanol	962.9 ± 0.023 ^f^	1.57 ± 1.20 ^d^	38.97 ± 0.83 ^c^	200.44 ± 0.02 ^b^
S6	Methanol	446.0 ± 0.06 ^c^	1.82 ± 0.76 ^f^	43.12 ± 0.92 ^d^	303.40 ± 0.03 ^c^
S7	Water	1305.6 ± 0.03 ^g^	0.55 ± 0.84 ^b^	81.27 ± 0.77 ^g^	962.66 ± 0.02 ^e^
	scCO_2_ + EtOH				
1	15 MPa:40 °C	1221.6 ± 0.075 ^a^	0.28 ± 0.73 ^c^	89.66 ± 0.74 ^b^	976.74 ± 0.01 ^d^
2	15 MPa:80 °C	1158.2 ± 0.01 ^ab^	0.34 ± 1.25 ^d^	84.60 ± 0.29 ^a^	848.59 ± 0.02 ^a^
3	25 MPa:40 °C	1249.0 ± 0.01 ^a^	0.18 ± 2.31 ^b^	93.62 ± 0.37 ^c^	903.41 ± 0.01 ^c^
4	25 MPa:80 °C	1391.9 ± 0.09 ^c^	0.17 ± 0.79 ^a^	94.09 ± 0.32 ^c^	984.89 ± 0.01 ^e^
5	20 MPa:60 °C	1058.9 ± 0.02 ^a^	0.34 ± 1.20 ^d^	89.94 ± 0.24 ^b^	884.89 ± 0.01 ^b^

Different superscripts indicate a significant difference (*p* < 0.05). * The values found were outside the confidence interval studied; thus, they are not shown.

**Table 5:** Results of the identification of phenolic contents by high-performance liquid chromatography.

				Phenolic Compounds Identified (μg g^−1^)							
Run (M_RM_)	Solvent	*p* (MPa)	T (°C)	Rt (min)	Gallic Acid	Rt (min)	Trans-Cinnamic Acid	Rt (min)	Genistein	Rt (min)	Daidzein
1	CO_2_+ EtOH	15	40	3.46	416.3 ^a^	25.46	518.3 ^b^	26.06	593.1 ^a^	24.59	606.1 ^c^
2	CO_2_+ EtOH	15	80	3.42	449.5 ^b^	25.37	521.4 ^c^	25.94	625.3 ^d^	24.50	605.0 ^c^
3	CO_2_+ EtOH	25	40	3.42	414.5 ^a^	25.36	507.0 ^a^	25.94	596.5 ^b^	24.50	577.3 ^a^
4	CO_2_+ EtOH	25	80	3.42	457.2 ^c^	25.38	518.1 ^b^	25.95	621.7 ^c^	24.50	603.9 ^c^
5	CO_2_+ EtOH	20	60	3.42	417.7 ^a^	25.38	518.7 ^b^	25.95	597.6 ^b^	24.51	565.9 ^b^

Different superscripts indicate a significant difference (*p* < 0.05).

## Data Availability

Not applicable.
